# Research note: Nutritive value of cold-pressed flaxseed meal for broiler chicken

**DOI:** 10.1016/j.psj.2024.103535

**Published:** 2024-02-07

**Authors:** Protus W. Nyende, Tofuko A. Woyengo

**Affiliations:** ⁎ Department of Agricultural, Food and Nutritional Science, University of Alberta, Edmonton, Alberta, Canada T6G 2P5; † Department of Animal Science, South Dakota State University, Brookings, SD, 57007, USA; ‡ Department of Animal Science, Aarhus University, Tjele, DK-8830, Denmark

**Keywords:** amino acid digestibility, apparent metabolizable energy, broiler, cold-pressed flaxseed meal

## Abstract

A study was conducted to evaluate standardized ileal digestibility (**SID**) of amino acids (**AA**) and N-corrected apparent metabolizable energy (**AMEn**) values of cold pressed flaxseed meal (**CPFM**) for broilers. One hundred and twenty broiler chicks were divided into 20 groups of 6 birds/group and fed 2 diets in a completely randomized design (10 groups/diet) from 14 to 21 d of age. The diets were cornstarch-based containing CPFM or conventional soybean meal (**SBM**; reference feedstuff) as the sole protein source. A N-free diet fed in another study conducted in the same facility and at the same time that the current study was conducted was used to estimate basal endogenous AA losses, and to calculate gross energy retention by difference method. The CPFM had greater neutral detergent fiber and ether extract contents (21.40 vs. 8.18% and 20.4 vs. 2.47% as is, respectively), but lower CP (20.47 vs. 48.28% as is) than SBM. The SID values of all indispensable AA (except for Leu, Phe and Trp) for CPFM were lower (*P* < 0.05) than those for SBM. The apparent retention of gross energy (65.95 vs. 44.24%) and N (55.53 vs. 9.79%), and AMEn (2,699 vs. 2,491 kcal/kg) for CPFM were lower (*P* < 0.05) than those for SBM. In conclusion, CPFM can serve as alternative oilseed co-product feedstuff for poultry. However, the CPFM has lower SID of AA and AMEn values than SBM likely due to the greater fiber content in the former than in the latter.

## INTRODUCTION

Flaxseed meal, which is the co-product of oil production from flaxseed, has high crude protein (**CP**) content (> 20%; Araujo et al., 2022), and hence it can potentially be a good source of amino acids (**AA**) and energy (if it has a high content of remaining oil) in diets for poultry. Oil is extracted from oilseeds such as flaxseed mainly by 2 methods: 1) expeller pressing followed by solvent extraction and 2) expeller pressing alone. The expeller pressing followed by solvent extraction method is more efficient method of oil extraction than expeller pressing alone, and hence the former method of extraction yields oilseed meal that has low oil content but higher content of other meal components such as AA (Woyengo et al., 2016a). Also, the amount oil (and hence energy) and AA availability in expeller pressed meal varies depending on the amount of pressure that is applied to the seeds during oil extraction. The oil content in expeller pressed meal produced by pressing seeds at low pressure to ensure that the extraction temperature is around 50°C (cold pressing) is greater than that in expeller pressed meal produced by applying a lot of pressure and hence heat (Youn et al., 2015). The AA digestibility of expeller pressed meal produced by pressing seeds at high pressure may be lower than that of expeller pressed meal produced by processing seeds at low pressure due to heat damage of protein in expeller pressed meal produced by pressing seeds at high pressure. Thus, the energy value and AA digestibility of oilseed meals can vary depending on the oil extraction method. The energy retention and AA digestibility values of expeller pressed flaxseed meal produced by processing seeds at high pressure (**HPFM**) for poultry have been reported (Araujo et al., 2022). However, the AMEn and SID of AA values of cold-pressed flaxseed meal (**CPFM**) for poultry have not been reported.

Soybean meal (**SBM**) that is the most common source of AA in poultry is produced from soybean seeds by expeller pressing followed by solvent extraction, and hence it has low oil content. It was hypothesized that the AMEn value of CPFM is greater than of SBM for poultry because of the greater oil content in the former than in the latter, but CPFM and SBM are similar regarding SID of AA. The objective was to determine the SID of AA and AMEn values of CPFM in comparison with SBM for broilers.

## MATERIALS AND METHODS

Experimental procedures were reviewed and approved by the Institutional Animal Care and Use Committee at South Dakota State University (18-087E).

### Experimental Ingredients and Diets

Diets included cornstarch-based diets with SBM or CPFM as the sole source of AA (Table 1). The CPFM fed in this study was sourced from Stengel Oils (Milbank, SD). The CPFM had been produced by processing flaxseed at less than 54°C (barrel temperature). The SBM fed in this study was obtained from a local feed mill and was included as a reference in the study. The N-free diet fed in another study (Agyekum and Woyengo, 2022) conducted in the same facility and at same time that the current study was conducted was used to estimate basal endogenous AA losses for determining the SID of AA. The ratio of cornstarch to sugar, cellulose, and soybean oil in the test diets was identical to the N-free diet to allow calculation of energy retention of the test feedstuffs using the difference method. The diets contained titanium dioxide (0.3%) as an indigestible marker.

**Table 1:** Ingredient and analyzed nutrient composition of soybean meal (SBM)- and cold pressed flaxseed meal (CPFM)-based diets.^1^

Item, %	SBM	CPFM
Ingredient, as fed		
SBM	50.00	
CPFM		30.00
Cornstarch	37.50	54.10
Sucrose	3.84	5.53
Cellulose	1.44	2.08
Soybean oil	2.40	3.46
Limestone	0.98	0.98
Dicalcium phosphate	1.95	1.95
Vitamin premix^2^	0.25	0.25
Mineral premix^3^	0.25	0.25
Choline chloride	0.50	0.50
Salt	0.30	0.30
Chromic oxide^4^	0.30	0.30
Titanium dioxide	0.30	0.30
Analyzed nutrients, as fed		
Dry matter	93.74	94.70
Crude protein	22.55	8.40

1 The N-free diet fed in the study of Agyekum and Woyengo (2022) was used to estimate endogenous AA losses, and to serve as basal diet for calculating energy retention of the CPFM by difference method.

2 Provided the following per kg of diet: 50,094 IU vitamin A, 22,556 IU vitamin D3, 226 IU vitamin E, 15 mg thiamine, 33 mg riboflavin, 90 mg pantothenic acid, 10 mg folic acid, 301 mg niacin, 16 mg pyridoxine, 15 mg menadione, 90 mg cyanocobalamin, and 0.9 mg biotin.

3 Provided the following per kg of diet: 300 mg manganese, 50 mg iron, 40 mg copper, 275 mg zinc, 3.13 iodine, and 0.75 mg selenium.

4 We used titanium dioxide as the indigestibility marker in our laboratory for digestibility studies. However, we had issues with the equipment for analyzing titanium when starting this study. Therefore, we included chromic oxide to have the option of analyzing for chromium in case the equipment for titanium analysis was still not functional after our experiment.

### Birds, Housing, and Experiment Procedure

A total of 120 one-day-old male broiler chicks of Ross 308 strain were acquired from a commercial hatchery. The chicks were distributed to electrically heated Petersime battery brooders (Petersime Incubator Co., Gettysburg, OH) so that each cage (34 cm wide, 102 cm long and 24 cm high) housed approximately 14 birds. The room temperature followed the programs recommended for the Ross strain. Light was provided for 24 h daily throughout the experiment. Chicks were fed growth promoters-free commercial starter diet (3,050 kcal/kg ME, 22 % CP, 1.00% Ca, and 0.45% non-phytate P) from day 1 to 14 d of age. On d 14, the birds were redistributed into 20 cages (6 birds/cage) and group-weighed.

The 2 experimental diets were randomly assigned to the cages in a completely randomized design (10 cages per diet) and fed from d 14 to 21 of age. Fresh water and feed were given ad libitum throughout the experiment. On d 19 and 20, excreta samples were collected from each cage and stored frozen at –20°C for later laboratory analyses. On d 21, birds were euthanized via cervical dislocation, and ileum contents were gently squeezed out and stored frozen at –20°C for later laboratory analyses.

### Sample Preparation and Analyses

The collected excreta and ileal digesta samples were, respectively, pooled for each cage. The pooled excreta samples were oven-dried for 4 d at 60°C, whereas the pooled ileal digesta samples were freeze-dried. Thereafter, the dried excreta, ileal digesta, test feedstuffs, and diet samples were finely ground using a centrifugal mill (model ZM200; Retsch GmbH, Haan, Germany) to pass through a 0.75 mm screen. All samples were analyzed for DM, gross energy (**GE**), and CP (N × 6.25) as described in the study of Agyekum and Woyengo (2022). Samples were further analyzed as follows: test feedstuffs for neutral detergent fiber (**NDF**), ether extract (**EE**), and AA; diet and ileal digesta samples for AA and titanium content; and excreta samples for titanium content as described in the study of Agyekum and Woyengo (2022).

### Calculations and Statistical Analysis

Apparent ileal digestibility (**AID**) of AA, and apparent retention (**AR**) of N and gross energy for diets were calculated using the indicator method as described in the study of Agyekum and Woyengo (2022). The SID of AA for diets was calculated from AID of AA corrected for basal endogenous losses of AA also as described in the study of Agyekum and Woyengo (2022). The AID and SID of AA and N retention for the test feedstuffs were determined by the direct method. The energy retention for the test feedstuffs was determined by difference method as described by Fan and Sauer (1995). The AME values for test feedstuffs were calculated by multiplying GE by apparent retention of GE. The AMEn for test feedstuffs were calculated as described in the study of Agyekum and Woyengo (2022).

Data were subjected to analysis of variance using the GLM procedure of SAS (SAS version 9.3; SAS Inst. Inc., Cary, NC) for a completely randomized design. Treatment means (CPFM vs. SBM) were compared using the t-test procedure.

## RESULTS AND DISCUSSION

The analyzed composition of CPFM and SBM is presented in Table 2. The CP, AA, and NDF contents of SBM were within the range of values previously reported by Sauvant et al. (2004). The CP (28.41%) and NDF (21.40%) contents of the CPFM used in the current study were lower than the values previously reported for HPFM (30.9% CP and 23.4% NDF) by Sauvant et al. (2004), which could be attributed to the lower efficiency of oil extraction from flaxseed during the production of CPFM than during the production of HPFM. An increase in efficiency of extraction of oil from oilseeds leads to increased concentration (in resulting oilseed meals) of all non-oil components of oilseeds due to increased removal of oil that would otherwise dilute these non-oil components of the oilseeds. Indeed, the EE of the CPFM fed in the current study (20.47%) was greater than the value reported by Sauvant et al. (2004) for HPFM (8.1%). The CPFM had greater NDF and EE content and but lower CP and AA contents than in the SBM, which could be due to differences in methods used to produce these feedstuffs from their respective oilseeds as previously explained, and to species differences.

**Table 2:** Analyzed composition, standard ileal digestibility (**SID**) of amino acids, apparent retention of N and gross energy, and nitrogen-corrected metabolizable energy (**AMEn**) values for cold-pressed flaxseed meal (**CPFM**) and soybean meal (**SBM**).

Item	SBM	CPFM	SEM	*P* value
Analyzed composition, as fed basis, %				
Dry matter	92.70	93.90	-	-
Crude protein	48.28	28.41	-	-
Gross energy, kcal/kg	4,326	5,119	-	-
Ether extract	2.47	20.47	-	-
Neutral detergent fiber	8.18	21.40	-	-
Indispensable amino acids				
Arg	3.07	2.61	-	-
His	1.13	0.61	-	-
Ile	2.11	1.26	-	-
Leu	3.37	1.67	-	-
Lys	2.75	1.18	-	-
Met	0.61	0.56	-	-
Phe	2.23	1.31	-	-
Thr	1.65	1.05	-	-
Trp	0.63	0.46	-	-
Val	2.19	1.49	-	-
SID of indispensable amino acids, %				
Arg	93.32	91.97	0.379	<0.001
His	90.87	83.61	0.602	<0.001
Ile	88.73	86.77	0.596	0.041
Leu	88.25	87.13	0.600	0.216
Lys	91.10	87.36	0.570	0.001
Met	93.11	90.86	0.415	0.003
Phe	88.97	88.06	0.533	0.256
Thr	87.37	75.34	0.816	<0.001
Trp	90.78	95.27	0.439	<0.001
Val	87.96	83.25	0.861	0.004
Apparent retention, %				
N	55.58	9.79	1.360	<0.001
Gross energy	65.97	44.24	1.383	<0.001
AMEn, kcal/kg, DM	2,699	2,491	57.73	0.026

The SID of AA, AR of N and GE, and AMEn values for CPFM and SBM are presented in Table 2. The SID of AA for CPFM was lower than that of HPFM fed in the study of Araujo et al. (2022). As previously mentioned, HPFM compared with CPFM is subjected to more heat during oil extraction, and hence the SID of AA for HPFM is expected to be lower than that of CPFM due to heat damage of protein in the HPFM. However, it should be noted that the amount of pressure that is applied to oilseeds during cold-pressing may not be sufficient to rupture all cells within the oilseeds to release or increase the availability of cell wall encapsulated oil for extraction and of cell wall encapsulated nutrients for digestion as evidenced by the high EE in CPFM. The Lys to CP ratio for CPFM fed in the current study was similar to that for HPFM fed in the study of Araujo et al. (2022; 4.1 vs. 3.7%), indicating that extent of protein damage (if any) in CPFM fed in the current study was similar to that for HPFM fed in the study of Araujo et al. (2022). Thus, the greater SID of AA values for HPFM fed in the study of Araujo et al. (2022) than for CPFM fed in the current study could be attributed to the fact that the amount of pressure applied to the HPFM was high enough to release relatively more encapsulated nutrients, but was not too high to damage protein. Woyengo et al. (2016b) similarly reported greater SID of AA values canola meal that had produced by extracting oil from canola seeds at the conventional high pressure than for canola meal that had been produced by cold-pressing. Araujo et al. (2022) did not report the actual temperature at which HPFM fed in their study was produced. The SID values of all indispensable AA (except for Leu, Phe and Trp) were lower (*P* < 0.05) in birds fed CPFM than in those fed SBM, which could partly be due to the greater fiber content in CPFM than in SBM. Additionally, flaxseed contains soluble fiber (mucilage), which can reduce AA digestibility by increasing digesta viscosity (Agyekum and Nyachoti, 2017).

The AR of N for CPFM was lower (*P* < 0.05) than that for SBM, which was explained by the lower SID of AA for the former than for the latter. The AR of GE for CPFM was lower (*P* < 0.05) than that of SBM, which could be attributed to the lower AR of N for the former than for the latter. The AMEn value of SBM (2,699 kcal/kg) was close to values (2,271–2,667 kcal/kg, DM basis) previously reported by Bertechini et al. (2019) for SBM. The AMEn value of CPFM was lower than the value reported by Araujo et al. (2022) for HPFM (3,074 kcal/kg, DM basis), which could be explained by the lower nutrient digestibility of the former than of the latter as previously discussed. The AMEn for CPFM was lower (*P* < 0.05) than for SBM, and this was due to lower AR of N and GE values for the former than the latter.

In conclusion, the AMEn and SID of AA values of CPFM fed in the current study were lower than those of SBM, which was partly attributed to a greater fiber content in the former than the later. The CPFM can therefore serve as alternative oilseed co-product feedstuff for poultry provided the presence and effect of fiber on reducing the AMEn and SID of AA are considered during the diet formulation.
